# Genomics and proteomics approaches to the study of cancer-stroma interactions

**DOI:** 10.1186/1755-8794-3-14

**Published:** 2010-05-04

**Authors:** Flávia C Rodrigues-Lisoni, Paulo Peitl, Alessandra Vidotto, Giovana M Polachini, José V Maniglia, Juliana Carmona-Raphe, Bianca R Cunha, Tiago Henrique, Caique F Souza, Rodrigo AP Teixeira, Erica E Fukuyama, Pedro Michaluart, Marcos B de Carvalho, Sonia M Oliani, Head and Neck Genome Project GENCAPO, Eloiza H Tajara

**Affiliations:** 1 Department of Molecular Biology, School of Medicine (FAMERP), São José do Rio Preto, Brazil; 2 Department of Biology, Instituto de Biociências, Letras e Ciências Exatas (IBILCE), São Paulo State University (UNESP), São José do Rio Preto, Brazil; 3 Department of Otorhinolaryngology and Head and Neck Surgery, School of Medicine (FAMERP), São José do Rio Preto, Brazil; 4 Department of Genetics and Evolutionary Biology, Institute of Biosciences, University of São Paulo (USP), São Paulo, Brazil; 5 Department of Head and Neck Surgery, Arnaldo Vieira de Carvalho Hospital, São Paulo, Brazil; 6 Division of Head and Neck Surgery, Department of Surgery, School of Medicine (USP), São Paulo, Brazil; 7 Department of Head and Neck Surgery, Heliópolis Hospital, São Paulo, Brazil; 8 Department of Biology and Zootechny, Faculty of Engineering of Ilha Solteria (UNESP), Ilha Solteira, Brazil; 9 Author list and addresses presented in the Acknowledgements

## Abstract

**Background:**

The development and progression of cancer depend on its genetic characteristics as well as on the interactions with its microenvironment. Understanding these interactions may contribute to diagnostic and prognostic evaluations and to the development of new cancer therapies. Aiming to investigate potential mechanisms by which the tumor microenvironment might contribute to a cancer phenotype, we evaluated soluble paracrine factors produced by stromal and neoplastic cells which may influence proliferation and gene and protein expression.

**Methods:**

The study was carried out on the epithelial cancer cell line (Hep-2) and fibroblasts isolated from a primary oral cancer. We combined a conditioned-medium technique with subtraction hybridization approach, quantitative PCR and proteomics, in order to evaluate gene and protein expression influenced by soluble paracrine factors produced by stromal and neoplastic cells.

**Results:**

We observed that conditioned medium from fibroblast cultures (FCM) inhibited proliferation and induced apoptosis in Hep-2 cells. In neoplastic cells, 41 genes and 5 proteins exhibited changes in expression levels in response to FCM and, in fibroblasts, 17 genes and 2 proteins showed down-regulation in response to conditioned medium from Hep-2 cells (HCM). Nine genes were selected and the expression results of 6 down-regulated genes (*ARID4A*, *CALR*, *GNB2L1*, *RNF10*, *SQSTM1*, *USP9X*) were validated by real time PCR.

**Conclusions:**

A significant and common denominator in the results was the potential induction of signaling changes associated with immune or inflammatory response in the absence of a specific protein.

## Background

Solid tumors are characterized by the presence of two major components: neoplastic cells and a specialized nonmalignant stroma in which they are immersed and are essential for their survival and proliferation. In carcinomas, a basement membrane is usually present between these components [1,2].

The tumor stroma is distinguished by an enrichment of microvessel density, abundance of endothelial cells and precursors, inflammatory cells including lymphocytes, neutrophils, macrophages, dendritic and mast cells, and a connective tissue with fibroblasts, myofibroblasts and histiocytes responsible for remodeling and deposition of extracellular matrix (ECM) components - fibronectin, collagens, elastin, and glycosaminoglycans [2-4]. Although these cells are nonmalignant, they have a unique gene expression pattern, compared to stroma cells in normal tissues [5,6].

Substantial evidence indicates that the development and the progression of cancer not only depend on its genetic characteristics but also on interactions with its microenvironment [4,7,8]. In fact, tumor cells may alter the surrounding stroma through direct cell contact or via the secretion of paracrine soluble factors, inducing cell differentiation or extracellular matrix modifications [9]. In it turn, stromal cells may promote cancer progression and acquisition of invasiveness [10-12]. It is possible that such interactions contribute to the neoplastic cell phenotype and behavior as observed during the normal development process and function of organs and tissues [13,14]. As Albini and Sporn (2008) appropriately propose, the microenvironment may be more than a partner but also an essential component of the cancer, and both should be considered as a functional whole [15].

In this context, inflammation and infection have gained special attention. Well known examples connecting infection-related or -unrelated chronic inflammation and increased risk for cancer development are described in the literature [16], and probably more than 15% of cancers are linked to these factors [17]. TNF-alpha and NF-κB transcription factor should play a central role in this process, modulating transcription of genes encoding angiogenic and growth factors, inflammatory cytokines and anti-apoptotic proteins [16]. In fact, many inflammatory mediators may influence cell proliferation and tumor development, as demonstrated by our recent studies on annexin A1 [18-20].

Macrophages represent one of the main inflammatory regulators in tumor stroma and are responsible for proliferation, invasion and immunosuppressive signaling, with the production of angiogenic and growth factors, chemokines, cytokines and matrix metalloproteinases [21]. The key partners of macrophages in this network are fibroblasts, the so-called carcinoma-associated fibroblasts (CAFs), which significantly increase the growth of neoplastic or normal cells [22,23] and can enhance tumor engraftment and metastasis in animal models [24]. Recently, Hawsawi et al. (2008) [25] observed well-defined differences in gene expression and proteomic profiles between activated CAFs and fibroblasts from normal stroma, emphasizing their importance in the cancer process.

Regardless of the fact that they are easily identified by their morphology, specific cellular markers for fibroblasts remain unknown, presumably because of their large diversity [26]. In tumor stroma, fibroblasts present a phenotype similar to those associated with wound healing, with a large and euchromatic nucleus and prominent rough endoplasmic reticulum [27,28]. These signals mediating the transition of normal to reactive fibroblasts are still not completely defined.

Many studies have analyzed the role of fibroblasts in cancer initiation and progression. To address this issue, several approaches have been used, as co-culture of cancer cells and fibroblasts and cultures with conditioned medium, combined or not with *in vivo *experiments. The data have shown that these cells, similar to macrophages, overexpress chemokines, interleukines, growth factors and matrix metalloproteinases, promoting inflammatory responses and facilitating angiogenesis, cancer-cell invasion and proliferation [29-31]. In head and neck cancer, for example, *in vitro *experiments have suggested that the presence of fibroblasts is essential for invasive features either because cancer cells express higher levels of matrix metalloproteinases in the presence of fibroblasts [32,33] or because cancer-associated fibroblasts themselves synthesize these proteins [34,35].

Much of the answer to the question of tumor-stroma interactions lies in the identity of ligands, receptors and effectors of signaling patterns expressed by stroma and tumor cells. Numerous growth factors, cytokines, chemokines, hormones, enzymes and cells responsible for their expression have been characterized but the cross-signaling between pathways in this complex network is far from solved [7,36]. Adding complexity to the scenario, the chemomechanical environment of the extracellular matrix may also act in concert with signaling pathways and affect the cancer process [37].

An important perspective in the study of tumor stroma is the potential use of the gene expression pattern of their cells for diagnostic or prognostic evaluation and as a target for therapy. Supporting this idea are the results from studies on outcome prediction and molecular marker analysis of the stroma [6,38], drugs targeting inflammatory cells [39] and mediators of angiogenesis [40,41].

In order to investigate potential mechanisms by which the tumor microenvironment might contribute to cancer phenotype, we asked whether soluble paracrine factors produced by stromal and neoplastic cells *in vitro *may influence proliferation, and gene and protein expression. For these purposes, we exploited purified fibroblasts isolated from a primary oral cancer and an epithelial cancer cell line linked by conditioned medium and genomic and proteomic approaches. Both cells were treated with the conditioned medium of each other and submitted to analysis by rapid subtraction hybridization methodology, two-dimensional electrophoresis and mass spectrometry. Based on the results of the rapid subtraction hybridization (RaSH) approach, a comparative quantitative real-time PCR was performed to validate the expression of several genes, focusing on those involved in tumorigenesis and inflammation. The results pointed to the participation of several inflammatory mechanisms that might have biological significance in epithelial tumors.

## Methods

### Primary tumor samples

For conditioned medium experiments, a primary epidermoid (squamous cell) carcinoma of the retromolar area was obtained from a 49-year-old male patient, prior to radiation and/or chemotherapy. Twenty-four laryngeal and 23 oral tongue squamous cell carcinoma (SCC) samples from patients undergoing tumor resection were used for gene expression analysis. All carcinoma samples were reviewed by senior pathologists and exhibited the presence of at least 70% tumor cells; the corresponding surgical margins were classified to be free of tumor cells.

The study protocol was approved by the National Committee of Ethics in Research (CONEP 1763/05, 18/05/2005), and informed consent was obtained from all patients enrolled.

### Epithelial cancer cell line and primary tumor cell cultures

The Hep-2 cell line, originally established from an epidermoid carcinoma of the larynx (ATCC, Rockville, Maryland, USA), was seeded at a density of 1 × 10^6 ^cells/mL per 75 cm^2 ^culture flask (Corning, NY, USA) in medium MEM-Earle (Cultilab, Campinas, SP, Brazil), pH 7.5, supplemented with 20% fetal calf serum (Cultilab), 1% non-essential amino acids, 0.1% antibiotic/antimycotic (Invitrogen Corporation, Carlsbad, CA, USA), and cultured at 37°C in a humid atmosphere of 5% CO_2_.

A primary carcinoma of retromolar area sample showing epithelium and adjacent connective tissues was rinsed multiple times with 100× antibiotic and antimycotic solutions (Invitrogen) and minced into 2-4 mm fragments. Single-cell suspensions were obtained by digestion at 37°C for 1 hour with 40 mg/mL collagenase type I (Sigma Chemical, St Louis, USA). After centrifugation, the cells were washed with PBS, resuspended in DMEM medium supplemented with 20% fetal calf serum (Cultilab), 2 mM glutamine (Invitrogen), 1% non-essential amino acids (Invitrogen), and 0.1% antibiotic/antimycotic (Invitrogen). The cells were seeded at a density of 1 × 10^6 ^cells/mL per 75 cm^2 ^culture flasks (Corning) and cultured at 37°C in a humid atmosphere of 5% CO_2_. Cell medium was changed at 72 h intervals until the cells became confluent. Since fibroblasts were mixed with the epithelial tumor cells at the time of initial plating, fibroblasts were selected by plating the cells growing in medium supplemented with 20% serum for at least 3 weeks [42-44].

### Preparation of conditioned medium

Conditioned medium (CM) was prepared from Hep-2 cell or tumor stromal fibroblast cultures showing 80% confluence. Twenty-four, 48 and 72 hours after medium replacement, the supernatant or conditioned medium (CM24, CM48 and CM72, respectively) from three replicas was aspirated and filtered through a 0.22 μm membrane (Millipore) to remove any cell debris and stored at -80°C. Before using, the CM was diluted 1:1 in complete medium. The dilution 1:1 and CM72 were chosen to maximize the chance of detecting a cell response to soluble factors. Optimization experiments showed that dilutions lower than 1:1 resulted in higher numbers of dead cells.

Hep-2 cell-conditioned medium is referred to as HCM and fibroblast-conditioned medium is referred to as FCM.

### Growth curve

Hep-2 cells were seeded at a density of 5 × 10^4 ^cells in plastic 6-well plates in two sets of quadruplicates. Twenty-four hours later, when cells had already adhered, Hep-2 cultures were incubated with FCMs. One replica in each set was treated with self-conditioned medium and one replica was treated with complete medium.

Medium was replaced on day 4 and cell morphology was observed every day. After 1, 3, 5 and 7 days, cells were harvested and counted using a Neubauer hemocytometer. The same experiment was repeated twice.

### Immunofluorescence analysis

The Hep-2 cell line or tumor stromal fibroblasts were grown in culture chambers (Nunc, Naperville, IL, USA) and, after 3 days, the chambers were carefully removed, and the slides with adherent cells were fixed in 4% paraformaldehyde and 0.5% glutaraldehyde, 0.1 mol/L sodium phosphate buffer, pH 7.4, for 2 hours at 4°C. The slides were washed in the same buffer and incubated with 0.1% albumin bovine and 3% normal serum in PBS (PBSA) to block nonspecific binding. The cells were immunostained with primary mouse monoclonal antibodies (Ab) anti-vimentin (NCL-VIM-V9, Novocastra, Benton Lane, Newcastle, UK) or anti-cytokeratin (M3515, antibodies to all types of cytokeratins; AE1-AE3; Dako, Carpinteria, CA, USA) diluted at 1:200 in 1% PBSA, followed by overnight incubation at 4°C. For negative controls, the cells were incubated with nonimmune mouse serum (1:200 working dilution; Sigma-Aldrich). After repeated washings in 1% PBS, a goat anti-mouse IgG (Fc fragment-specific, Dako, Glostrup, Denmark) antibody conjugated to FITC (1:50; British BioCell International, Cardiff, UK) was added, followed by 1 hour incubation at room temperature. Thus, the cells were washed thoroughly in PBS. Analysis was conducted using an Axioskop 2 light microscope (Zeiss, GR) equipped with a digital camera. Digital images were captured by using software AxioVision (Zeiss, GR).

### Immunohistochemical analysis

Apoptosis was assayed using AnxA5 staining as described [45]. Fixed Hep-2 cell line or tumor stromal fibroblast in slides from culture chambers were incubated with the following reagents: 2.1% sodium citrate for 30 min at 96°C; 3% hydrogen peroxide for 15 min; 0.1% Tween 20 (Sigma-Aldrich) diluted in 0.4% PBS for 15 min; non-specific binding sites were blocked with 10% albumin bovine (BSA) diluted in TBS (20 mM Tris buffer in 0.9% NaCl, pH 8.2) for 30 min. The slides were then incubated overnight with a rabbit polyclonal antibody anti-AnxA5 (sc8300, Santa Cruz Biotechnology, California, USA), diluted 1:200. After repeated washings in 1% PBSA, a goat anti-rabbit IgG (Fc fragment specific) antibody conjugated to 5 nm colloidal gold particles (N24916, Invitrogen) was added. Silver enhancing solution (L24919, Invitrogen) was used to augment gold particle staining. At the end of the reaction, cells were washed thoroughly in distilled water, counterstained with haematoxylin and examined using an Axioskop2 microscope (ZEISS, GR).

### RNA extraction for Rapid Subtraction Hybridization (RaSH) and real time PCR experiments

Hep-2 cells and stromal fibroblasts were seeded at a density of 1 × 10^6 ^cells/mL per 75 cm^2 ^culture flasks in complete medium (controls) and in conditioned medium. Hep-2 cells and fibroblasts were cultured for 5 and 3 days, respectively, and harvested by addition of TRIzol Reagent, following treatment with DNase (Invitrogen). Total RNA from primary tumor samples was also extracted using TRIzol Reagent and treated with DNase. cDNA synthesis was performed using a High Capacity cDNA Archive kit (Applied Biosystems, Foster City, CA, USA) as described by the manufacturer.

### RaSH

RaSH technique was performed as described by Jiang et al. (2000) [46]. Aliquots (20 μg) of total RNA from control cells (driver) or treated cells (tester) were used for double-stranded cDNA synthesis using standard protocols [47].

The cDNA was digested with MboI (Invitrogen) at 37°C for 3 h followed by phenol/chloroform extraction and ethanol precipitation. The digested cDNAs were mixed with the adaptors XPDN-14 5'-CTGATCACTCGAGA and XPDN-12 5'-GATCTCTCGAGT (Sigma Chemical, final concentration 20 μM) in 30 μl of 1× ligation buffer (Gibco BRL), heated at 55°C for 1 min, and cooled down to 14°C within 1 h. After adding 3 μl of T4 DNA ligase (5 U/μl) (Gibco, BRL), ligation was carried out overnight at 14°C. After phenol/chloroform extraction and ethanol/glycogen precipitation, the mixtures were diluted to 100 μl with TE buffer (10 mM Tris/1 mM EDTA); 40 μl of the mixtures were used for PCR amplification.

The PCR mixtures were set up using 10 μM XPDN-18 5'-CTGATCACTCGAGAGATC, 0.4 mM dNTPs, 10 × PCR buffer, 1.5 mM MgCl_2 _and 1U Taq DNA polymerase (Invitrogen). Thermocycler conditions were one cycle at 72°C for 5 min, followed by 25 cycles of 94°C for 1 min, 55°C for 1 min, 72°C for 1 min, ending in a final extension at 72°C for 3 min. Ten μg of purified PCR product (tester) was digested with 20U XhoI (Invitrogen) followed by phenol/chloroform extraction and ethanol precipitation.

One-hundred nanograms of the tester cDNA were mixed with 5 μg of the driver cDNA in hybridization solution (0.5 M Nacl, 50 mM Tris/HCl, SDS2% and 40% formamide) and, after heating at 95°C, incubated at 42°C for 48 h. After extraction and precipitation, the hybridization mixture (1 μg) was ligated with XhoI-digested pZero plasmid and transformed into competent bacteria. Bacterial colonies were picked and used as DNA template for PCR. Clones were sequenced using an automated DNA sequencer and sequence homologies were searched using the BLAST program [48]. Gene ontology (GO) annotation was used for the functional classification of up- and down-regulated genes [49].

### Quantitative PCR

For validation experiments, cells were seeded at a density of 1 × 10^6 ^cells/mL per 75 cm^2 ^culture flasks in two sets of quadruplicates. Twenty-four hours later, when cells had already adhered, Hep-2 culture replicas were treated with FCMs and fibroblast cultures were treated with HCMs. One replica in each set (control) was treated with self-conditioned medium. Hep-2 cells and fibroblasts were harvested after 5 and 3 days, respectively, and RNA was extracted as described above.

Nine differentially expressed genes were selected for validation by quantitative real time PCR experiments according to their direct or indirect involvement in tumorigenesis. Their expression was checked in treated samples relative to matched non-treated samples. One of these genes (*ARID4A*) was also selected for quantitative real time PCR validation in fresh tumor samples of 24 laryngeal SCC and in 23 oral tongue SCC relative to matched normal samples.

The primers were manually designed with: 19-23 bp length, 30-70% GC content and a short amplicon size (90-110 bp). Their sequences are available upon request. Real time PCR was performed in triplicate using a 7500 Fast Real-Time PCR System (Applied Biosystems). Reaction mixture consisted of a 20 ul volume solution containing 10 ul of Power SYBR Green PCR Master Mix (Applied Biosystems), 500 nM of each primer and 100 ng cDNA. The PCR conditions were 95°C for 10 min followed by 40 cycles of 95° for 15 s and 60° for 1 min. Melting curve analysis was performed for each gene to check the specificity and identity of the RT-PCR products.

For each primer set, the efficiency of the PCR reaction (linear equation: y = slope + intercept) was measured in triplicate on serial dilutions of the same cDNA sample. The PCR efficiency (E) was calculated by the formula *E *= [10^(-1/slope)^] and ranged from 1.96 to 2.02 in the different assays.

Three control genes (*GAPDH, ACTB *and *TUBA6*) were used as internal standards. The relative expression ratio (fold change) of the target genes was calculated according to Pfaffl (2001) [50]. Statistical analysis was performed by a two-tailed unpaired *t *test using GraphPad prism software.

### Proteomic analysis

Hep-2 cells and stromal fibroblasts were seeded at a density of 1 × 10^6 ^cells/mL per 75 cm^2 ^culture flasks in complete medium and in conditioned medium, as described for RASH experiments. Hep-2 cells and fibroblasts were cultured for 5 and 3 days, respectively, and harvested by centrifugation at 3200 rpm for 5 min at 4°C. Cells were disrupted by sonication, proteins were isolated and two-dimensional electrophoresis (2-DE) was performed, as described by de Marqui et al. (2006) [51]. Briefly, isoelectric focusing was carried out in a IPGphor (GE Healthcare) using 13-cm immobilized pH 3-10 L gradient strips. Vertical 12.5% SDS-PAGE was performed in a SE 600 Ruby electrophoresis unit (GE Healthcare) and proteins were detected by Coomassie Blue staining. Differentially expressed proteins were excised from gel, distained, dried and in-gel tryptic-digested. Negative and positive control digests were performed on gel slices that contained no protein and on slices cut from a band of the molecular weight marker, respectively.

Samples were analyzed using MALDI Q-TOF (Matrix Assisted Laser Desorption Ionization - Quadrupole Ion Filter - Time of Flight) Premier (Waters Corporation, Milford, MA, USA) mass spectrometer (MS/MS). Duplicate or triplicate runs of each sample were made to ensure an accurate analysis.

For protein identification, the resulting MS/MS data were interpreted by MASCOT software (MS/MS Ions Search) [52] and searched against the Mass Spectrometry Protein Sequence Database (MSDB). The UniProtKB/Swiss-Prot [53] database was used for the functional classification of up- and down- expressed proteins.

### Data Handling and Statistical Analysis

Quantification of apoptotic cells was performed with a high magnification objective (×40) counting cells in 100 μm^2 ^areas and reported as mean ± SEM per group. Densitometric analysis for the immunofluorescence staining used an arbitrary scale ranging from 0 to 255 units. Statistical differences between groups were determined by analysis of variance followed, if significant, by the Bonferroni test.

## Results

### Stromal fibroblasts: selection and immunofluorescence analysis

**Figure 1:**
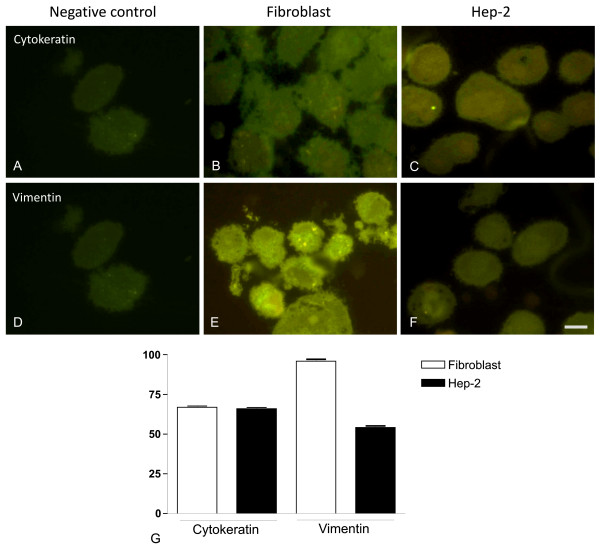
**Immunofluorescence analysis of cytokeratin and vimentin in stromal fibroblasts and Hep-2 cell line**. (A and D) Absence of immunoreactivity in sections incubated with control nonimmune mouse serum. Stromal fibroblasts (B and E) and Hep-2 cell line (C and F) were positive for vimentin and cytokeratin, respectively. (G): Densitometric analysis of immunofluorescence reaction to vimentin and cytokeratin in stromal fibroblasts and Hep-2 cell line. Scale bar, 20 μm.

Fetal calf serum concentration and culture time provided a simple method of selecting fibroblasts from a primary carcinoma of retromolar area. Fibroblast cultures at passage 78 still showed spindle-shaped cells, which displayed the typical fibroblast markers, weak cytokeratin and intense vimentin immunoreactivity in cytoplasm, after immunofluorescence analysis (Figure 1B, E). Staining was obtained with both antibodies (cytokeratin and vimentin) in Hep-2 cells (Figure 1C, F). No labeling was detected in sections incubated with the control nonimmune mouse serum (Figure 1A, D).

Ultrastructural analysis showed that the stromal fibroblasts present large euchromatic nuclei, more granular endoplasmatic reticulum, mitochondria and nucleoli than normal fibroblasts (data not shown). Therefore, the spontaneously immortalized cell line of fibroblasts retained the characteristics of stromal cells and may correspond to cancer-associated fibroblasts (CAF).

### Conditioned medium inhibits proliferation and induces apoptosis

**Figure 2:**
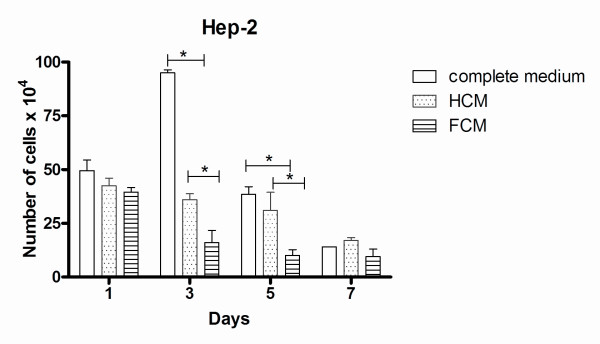
**Growth curve of Hep-2 cell line**. Hep-2 cells were cultured in complete medium, treated with self-conditioned medium (HCM) or with conditioned medium from fibroblast cultures (FCM) and collected 1, 3, 5 and 7 days after medium replacement. Data are means ± s.d. of two independent experiments in duplicates. *P < 0.05. Error bars indicate S.D.

Growth curves of Hep-2 cells treated with FCM showed decreased proliferation (Figure 2). Growth inhibition was observed as early as day 1 and was statistically significant (P < 0.05) at day 3 and day 5.

**Figure 3:**
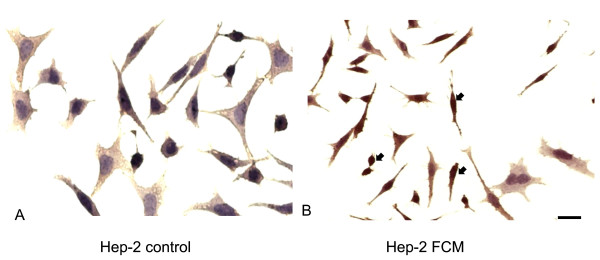
**Immunohistochemistry reaction with AnxA5 antibody showed the presence of gold particles on the cytoplasm of apoptotic cells**. Hep-2 cells (A) without treatment and (B) treated with conditioned medium from fibroblast culture (FCM) show AnxA5 immunoreactivity. Apoptotic cells immunolabeling for AnxA5 can be seen in Hep-2 cells treated with FCM (arrows). Staining with haematoxylin. Scale bar, 20 μm.

The immunohistochemistry reaction with AnxA5 antibody showed the presence of gold particles on the cytoplasm of the Hep-2 apoptotic cells (Figure 3). The AnxA5 immunoreactivity was found more in the apoptotic process of Hep-2 cells incubated in FCM (56%) than in cells without the treatment (24%). Apoptotic cells displayed distinctive morphology, a notable decrease in the nuclear size, irregular shape and cytoplasmic blebbing.

### Genes identified using the RaSH approach

A total of 81 clones from the Hep-2 cell line and fibroblast libraries were sequenced. In the Hep-2 cell line, forty-one genes exhibited changes in expression levels in response to FCM treatment (33 down- and 8 up-regulated) and, in fibroblasts, 17 genes showed down-regulation in response to HCM treatment. These genes are involved in response to stimulus, apoptosis, cell proliferation and differentiation, signal transduction, transcription, translation and transport (Table 1 and 2).

**Table 1:** Information on biological processes based on Gene ontology.

*Biological Process*	*Down-regulated genes*
**Cell communication**	
signal transduction	*FAS, SQSTM1, YWHAZ*

**Transcription**	*ARID4A, CALR, MYC, PARP1, RNF10, SQSTM1*

**Translation**	*AARS, RPLP0, RPS17, RPS23*

**Apoptosis**	*CALR*
induction	*FAS*
anti-apoptosis	*TPT1, YWHAZ*

**Cell migration**	*TMSB4X*

**Cell cycle**	*DYNC1H1, MYC, PSMC6*

**Cell proliferation**	
negative regulation	*GPNMB, LDOC1*
positive regulation	*MYC*

**Developmental process**	
epidermis development	*UGCG*

**Response to stimulus**	
defense response	
inflammatory response	*LTA4H*
response to stress	*EIF2AK1, SQSTM1*
response to oxidative stress	
response to external stimulus	*EIF2AK1*

**Transport**	*CALR, NDUFA4, SQSTM1*

**Metabolic process**	*COX7C, OLA1*,
protein metabolic process	*PARP1, SQSTM1, USP9X*
protein modification process	*GRPEL2, HSP90AB1, PPP2R2A, PRPF4B, USP48*
lipid metabolic process	*LTA4H, UGCG*
DNA repair	*PARP1*
RNA processing	*PRPF4B, SF3B1*

**Cellular homeostasis**	*CALR, MYC, RPS17*

**No classification**	*GNB2L1, RCN1*

	***Up-regulated genes***

**Transcription**	*ENO1*

**Translation**	*EIF1, TARS*

**Apoptosis**	*RTN3*
induction	*DAP3*

**Cell proliferation**	*PRDX1*
negative regulation	*ENO1*

**Developmental process**	
organ development	*PRDX1*

**Response to stimulus**	
response to stress	*EIF1, RTN3*

**Metabolic process**	*PRDX1*
protein metabolic process	
protein modification process	*P4HB*
nucleic acid metabolic process	
RNA processing	*USP39*

Top down- and up-regulated genes selected by RaSH in Hep-2 samples treated with FCM.

**Table 2:** Information on biological processes based on Gene Ontology.

*Biological Process*	*Down-regulated genes*
**Cell communication**	
signal transduction	*S100A6, FN1*

**Transcription**	*FOSL1*

**Translation**	*RPL37A, RPL7, RPL19, RPL27A, RPLP0*

**Apoptosis**	*CTSB*
anti-apoptosis	*TPT1*

**Cell adhesion**	FN1

**Cell proliferation**	
positive regulation	*S100A6, FOSL1*

**Developmental process**	
organ development	
epidermis development	*COL1A1*

**Response to stimulus**	
defense response	*FOSL1*
response to stress	*FN1*

**Transport**	*ERGIC3, STX4*

**Metabolic process**	
protein metabolic process	*CTSB*
RNA processing	*PRPF3*

**No classification**	*CIZ1, POLE4*

Top down-regulated genes selected by RaSH in CAF samples treated with HCM.

### Real-time PCR validation of differentially expressed genes

**Figure 4:**
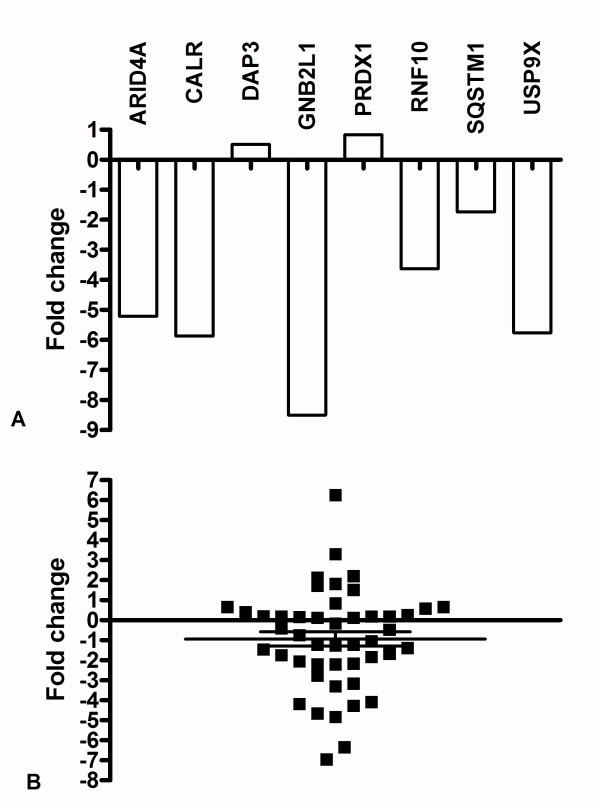
**Real-time PCR gene expression in a conditioned medium-treated neoplastic cell line and in primary tumors**. (A) Expression of *ARID4A, CALR, DAP3, GNB2L1, PRDX1, RNF10, SQSTM1 *and *USP9X *genes in Hep-2 cells treated with conditioned medium from fibroblast cultures. (B). *ARID4A *gene expression in 47 laryngeal and oral tongue carcinomas. Relative quantitation of target gene expression for each sample was calculated according to Pfaffl [50]; *GAPDH *was used as the internal reference and control sample as the calibrator. Values were Log2 transformed (y-axis) so that all values below -1 indicate down-regulation in gene expression while values above 1 represent up-regulation in tumor samples compared to normal samples.

Nine genes displaying down- (*ARID4A, CALR, GNB2L1, GPNMB, RNF10, SQSTM1, USP9X*) or up-regulation (*DAP3*, *PRDX1*) in Hep-2 cells treated with FCM were selected and the expression data for six down-regulated genes (*ARID4A, CALR, GNB2L1, RNF10, SQSTM1, USP9X*) were confirmed by real time PCR (Figure 4A). Most results were, therefore, consistent with the RaSH data.

*ARID4A *expression was also analyzed in 24 pairs of tumor and matched normal tissues from laryngeal squamous cell carcinomas and in 23 pairs of tumor and matched normal tissues from oral tongue squamous cell carcinomas. *ARID4A *mRNA levels were decreased (≥ 2-fold) in almost half of the squamous cell carcinomas samples (-1.04 to -6.9-fold change, 23 of 47 samples, i.e., 49%) and were increased in some of these samples (1.51 to 6.26-fold change, 7 of 47 samples, i.e., 15%) (Figure 4B). In contrast, no differences in transcript levels were observed between 17 of 47 samples (36%) and normal tissue. Therefore, similarly to the Hep-2 cell line, most primary head and neck tumors (49%) showed down-regulation of *ARID4A *transcripts.

No differences were observed in respect to clinicopathological features between samples presenting up- and down-regulation of *ARID4A *transcripts (Additional file 1).

### Proteomics approach

**Figure 5:**
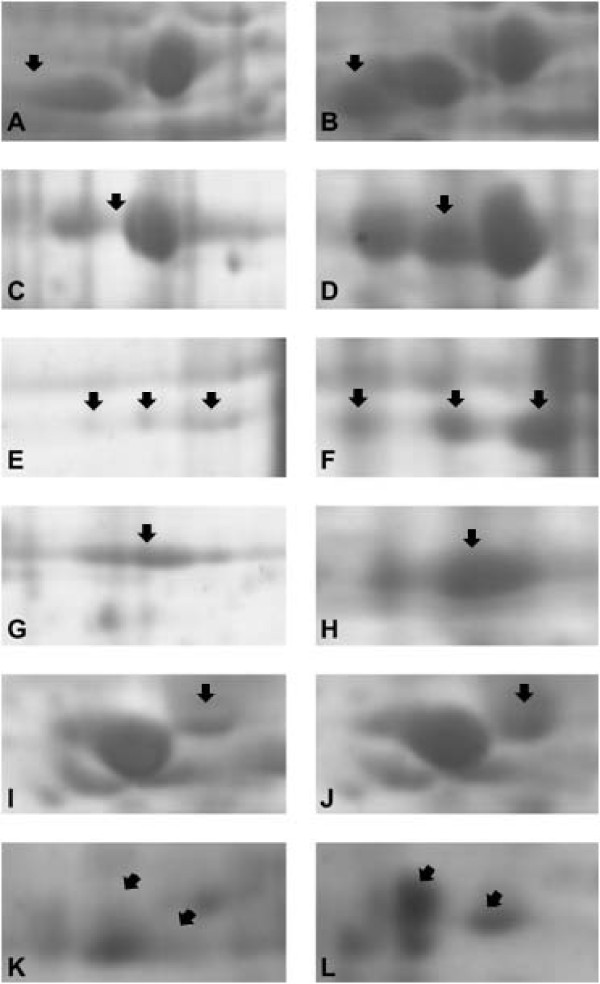
**Enlarged 2-DE gels of proteins from conditioned medium-treated Hep-2 cells and stromal fibroblasts**. Five proteins (arrows), tubulin beta (A-B), alpha enolase (C-D), aldolase A (E-F), glyceraldehyde-3-phosphate dehydrogenase (G-H) and heterogeneous nuclear ribonucleoprotein C (I-J) were down-regulated in Hep-2 cell line treated with fibroblast conditioned medium (A, C, E, G and I) and two proteins (K-L), vimentin (arrow on left) and actin (arrow on right), were underexpressed in fibroblasts treated with Hep-2 cell line conditioned medium (K).

Comparison between 2-DE patterns from treated cells and controls revealed approximately 80 spots with significant differences in intensity. Seven proteins (Figure 5) showing expression level changes in response to CM treatment were identified by MALDI-Q-TOF-MS mass spectrometry (Additional file 2). Five proteins (alpha enolase, heterogeneous nuclear ribonucleoprotein C C1/C2, aldolase A, tubulin beta and glyceraldehyde-3-phosphate dehydrogenase) were down-regulated in Hep-2 cell line treated with conditioned medium (FCM72) and two proteins (vimentin and actin) were underexpressed in fibroblasts treated with Hep-2 cell line conditioned medium (HCM72). These proteins are involved in transcription, growth control, response to stimulus, RNA processing, glycolysis, cell motion and membrane trafficking.

## Discussion

The molecular crosstalk between neoplastic and the surrounding tissue induces several stromal changes, including neoangiogenesis and immune/inflammatory reaction, as well as new extracellular matrix formation and the activation of fibroblast-like cells, a process known as desmoplasia [54], [55]. Initially, the desmoplastic response was considered a barrier against tumor invasion, but there is growing evidence that desmoplasia is an unfavorable prognostic factor. For example, Sis et al. [56] suggested that desmoplastia is related to increased risks of regional metastases, poorly differentiated primary tumors and lymphatic and venous invasion in colorectal carcinoma. Similar results were observed for head and neck squamous cell carcinomas, which show a high risk of neck recurrence in presence of a desmoplastic stromal pattern [57].

In the present study, we investigated the influence of soluble paracrine factors produced *in vitro *by stromal cells derived from an oral carcinoma and by a neoplastic epithelial cell line on proliferation and gene/protein expression. First, we noted that conditioned medium from stromal fibroblast cultures inhibited Hep-2 cell line proliferation and induced apoptosis, suggesting that factors secreted by fibroblasts include proteins that interfere in cell growth and death of neoplastic cells. In addition, using rapid subtraction hybridization and proteomic analysis, we identified gene products generated by stromal and neoplastic cells that may influence proliferation, differentiation and apoptosis, or drive response to stimulus.

Down-regulated genes in neoplastic cells treated with FCM are involved in signal transduction (*FAS*, *SQSTM1*, *YWHAZ*), transcription (*ARID4A*, *CALR*, *MYC*, *PARP1*, *RNF10*, *SQSTM1*), translation (*AARS*, *RPLP0*, *RPS17*, *RPS23*), apoptosis (*CALR*, *FAS*, *TPT1*, *YWHAZ*), cell migration (*TMSB4X*, *GNB2L1*), cell cycle and cell proliferation (*DYNC1H1*, *GPNMB*, *LDOC1*, *MYC*, *PSM*), epidermis development (*UGCG*), response to stimulus (*EIF2AK1*, *LTA4H*, *SQSTM1*), transport (*CALR*, *NDUFA4*, *SQSTM1*) and different metabolic processes (*USP9X*). Up-regulated genes are also involved in transcription and translation (*ENO1*, *EIF1*, *TARS*), apoptosis (*DAP3*, *RTN3*), cell proliferation (*PRDX1*, *ENO1*), organ development (*PRDX1*), response to stress (*EIF1*, *RTN3*) and metabolic processes (*PRDX1*, *P4HB*, *USP39*).

In fibroblasts treated with HCM, the biological processes of down-regulated genes include signal transduction (*S100A6*, *FN1*), transcription and translation (*FOSL1*, *RPL37A*, *RPL7*, *RPL19*, *RPL27A*, *RPLP0*), apoptosis (*CTSB*, *TPT1*), cell proliferation (*S100A6*, *FOSL1*), epidermis development (*COL1A1*), response to stimulus (*FN1*, *FOSL1*), transport (*ERGIC3*, *STX4*) and protein and RNA metabolism (*CTSB*, *PRPF3*).

Two genes exhibited similar patterns in both cells (*RPLP0*, *TPT1*), which may indicate that the transcript levels are affected by soluble paracrine factors produced by either fibroblasts or neoplastic cells or by other *in vitro *conditions. Therefore, they may not be specific to interactions between stroma and tumor.

After literature analysis, nine genes (*ARID4A*, *CALR*, *GNB2L1*, *GPNMB*, *RNF10*, *SQSTM1*, *USP9X*, *PRDX1 *and *DAP3*) showing potential involvement in signaling cascades related to tumorigenesis and/or stromal/tumor cell interactions were selected for validation by real-time RT-PCR using treated and non-treated cell lines. For six genes (*ARID4A*, *CALR*, *GNB2L1*, *RNF10*, *SQSTM1*, *USP9X*), the results were consistent with the RASH data. In almost half of the primary tumors analyzed, *ARID4A *transcripts also showed down-regulation, although no correlation with clinicopathological features was detected. These findings in primary tumors should reflect the complex network of a multi-cellular tissue, a situation contrasting with that of a neoplastic cell line cultured in medium conditioned by fibroblasts.

The product of *ARID4A *- AT rich interactive domain 4A (RBP1-like) - also known as *RBP1 *or *RBBP1 *gene, interacts with the tumor suppressor retinoblastoma (pRB) and histone-modifying complexes, repressing promoters of specific genes [58]. Röhl et al. [59] detected several genes, including *ARID4A*, overexpressed in astrocytes treated with medium conditioned by activated microglia, which protected them against stress conditions. Recently, Wu et al. [60] showed that *Arid4a*-deficient mice exhibit down-regulation of several homeobox genes and of the forkhead box gene *Foxp3*, which codes a transcription factor involved in the development and function of regulatory T cells [61]. These mice also show bone marrow failure with myelofibrosis and higher frequencies of hematologic malignancies, providing evidence that *ARID4A *functions as a tumor suppressor gene and its absence is permissive for the proliferation of connective tissue elements. The study of Perez et al. [62] added data on the role of this gene in cancer. These authors detected increased mRNA levels of *ARID4A *and *RB1 *in normal human epidermal keratinocytes treated with arsenic and benzo [a]pyrene *in vitro*. Since these chemicals alter proliferation and inhibit differentiation of keratinocytes [63-65], the findings may indicate that up-regulation of *ARID4A *is negatively related to epithelial differentiation. Therefore, the potential modulation of this gene by paracrine factors produced by stromal fibroblasts may represent an attempt to promote differentiation of neoplastic epithelial cells and, at the same time, their proliferation.

Calreticulin (coded by *CALR *or *CRT *gene) is a calcium-binding protein of the endoplasmic reticulum with intracellular and extracellular functions related to cellular adhesion, migration, and phagocytosis [66]. Calreticulin can be observed on the surface of stressed cells and, when bound to the plasma membrane of apoptotic cells, drives the phagocytosis by macrophages and dendritic cells [67]. In absence of this protein, the cells are not efficiently removed by phagocytes [68]. Recently, Nanney et al. [69] showed that calreticulin stimulates both migration and proliferation of keratinocytes and fibroblasts and apparently attracts monocytes and macrophages, suggesting its involvement in inflammatory response. Otherwise, fibroblasts underexpressing CARL exhibit weak adhesion and spreading [70]. Accordingly, Kypreou et al. [71] detected a correlation between calreticulin up-regulation and progression of fibrosis and also that TGF-beta, a contributing factor in fibrotic processes, up-regulated calreticulin in cultured human epithelial cells. In light of the data, we speculate that the low levels of this protein observed in treated Hep-2 cells inhibit proliferation, or represent a protective response of neoplastic cells to phagocytosis and antitumor immune process.

Guanine nucleotide binding protein (G protein), beta polypeptide 2-like 1 or Rack1 (coded by *GNB2L1 *gene) is a cytosolic protein homologous to the beta subunit of G proteins, and contains seven WD repeats, which act as sites for protein-protein interactions. Binding partners of GNB2L1 include protein kinase C, Src family kinases, components of the ERK pathway, cytokine and interferon receptors, beta integrins and many others. Many of these interactions are consistent with the participation of Rack1 in cell adhesion, movement and growth [72-75].

Sequestosome 1 or ubiquitin-binding protein p62 (coded by *SQSTM1 *or *p6*0 or *p62 *gene) is a 62-kDa protein that binds to the Src homology 2 (SH2) domain of p56^lck ^kinase in a phosphotyrosine-independent manner [76]. It has been suggested that p62 is a signaling adaptor which links different signal transduction pathways related to cell proliferation, differentiation and death, including NF-κB pathway [77-82]. *SQSTM1 *abnormal expression has been observed in hepatocellular, prostate and breast cancers [83-85] and is associated with poor outcomes in breast cancer [86].

Another gene down-regulated by fibroblast-conditioned medium is *USP9X *(ubiquitin specific peptidase 9, X-linked), also known as *DFFRX*, *FAF *or *FAM*. This gene is a member of the peptidase C19 family and encodes a protein similar to ubiquitin-specific proteases (USPs). These proteases regulate the production and recycling of ubiquitin and are critically involved in the control of cell growth, differentiation, and apoptosis [87]. Alteration of USPs may play an important role in the pathogenesis of cancer [88] and may exert distinct growth regulatory activities by acting as oncoproteins or tumor suppressor proteins. Overexpression of certain USPs correlates with progression towards a more malignant phenotype in carcinoma of lung, kidney, breast and prostate [89,90].

*RNF10 *(ring finger protein 10) is the least known gene selected for validation. The product contains a ring finger motif, which is involved in protein-protein interactions and has been described in proteins implicated in many cellular processes such as signal transduction, transcriptional regulation, ubiquination, and apoptosis [91,92].

With respect to proteomic analysis, few differences (mostly quantitative) between treated and non-treated cells were detected. Among the proteins differentially expressed, alpha-enolase, heterogeneous nuclear ribonucleoprotein C C1/C2, aldolase A, tubulin beta and glyceraldehyde-3-phosphate dehydrogenase were down-regulated in neoplastic cells treated with FCM and vimentin and actin were down-regulated in fibroblasts treated with HCM. These proteins, produced by neoplastic cells or fibroblasts, may affect tumorigenesis. For example, the glycolytic enzyme alpha-enolase and its enzymatically inactive isoform MBP-1 (c-*myc *promoter binding protein 1) are negative regulators for *MYC *expression [93,94]. *MYC *is one of the most frequently de-regulated oncogenes in cancer [95] and, in the absence of both enzymes, may become activated and accelerate tumor growth. Contrary to RaSH results, alpha enolase protein was observed underexpressed by proteomic analysis in treated Hep-2 cells, which may indicate a nonspecific finding or a post-transcriptional/posttranslational regulation of the RNA/enzyme.

## Conclusions

Fibroblasts, as other cells in tumor microenvironments, need to maintain close communication with cancer cells, promoting proliferation, recruitment of inflammatory cells and acquisition of invasive characteristics. Similarly, cancer cells may influence stromal cells to generate a favorable and supportive environment, which would supply them with nutrients and factors necessary for developing the tumor and spreading of metastasis. In the present study, we observed both positive and negative effects exerted by fibroblasts on Hep-2 cells, favoring or not the former. A significant and common denominator in the results was the potential induction of signaling changes associated with immune or inflammatory response in the absence of a specific protein. In fact, *ARID4A *down-regulation is related to low levels of the transcript factor Foxp3 [60], which in turn is linked to immune responsiveness by targeting NF-κB and CREB pathways [96]. The final effect is the inhibition of the inflammatory response and the cost is a permissive sign for fibroblast proliferation [60]. Down-regulation of *CARL *also blocks the inflammatory response but has negative effects on stroma growth [69]. In presence of low levels of Rack1, again a deficient or altered inflammatory response may occur since Rack1 underexpression has already been related to the deregulation of cytokine production [97]. Similar results have been observed in p62-deficient mice, which exhibit abnormal control of NF-κB activation and reduced inflammation in experimental conditions [98]. The opposite effect is expected for osteoactivin underexpression because this protein has been observed as a negative regulator of macrophage inflammatory responses [99].

The complexity of the tumor microenvironment is immense and much information is still necessary for better understanding how the relationship between stroma and carcinoma cells can be used for diagnostic and prognostic evaluation and a target for therapy.

## Competing interests

The authors declare that they have no competing interests.

## Authors' contributions

FCR-L participated in the design of the study and analysis of the data, carried out cell culture, RaSH experiments and drafted the manuscript. PPJr helped with RaSH experiments. AV and GMP carried out proteomics analysis. JVM was responsible for sample collection and processing. JC-R carried out cloning and sequencing of the samples. BRC carried out cell culture experiments. TH helped with manuscript preparation. CFS performed the real time PCR experiments. RAPT and SMO carried out immunofluorescence and immunohistochemical analysis. EEF and PMJr carried out clinical data analysis for sample selection. MBdC carried out clinical data analysis for sample selection and drafted the manuscript. GENCAPO team members were responsible for sample collection and initial on-site sample processing, provided the pathological analysis of the cases, obtained the informed consent and discussed the findings. EHT participated in the study design and coordination, carried out the analysis and interpretation of the data and drafted the manuscript. All authors read and approved the final manuscript.

## Pre-publication history

The pre-publication history for this paper can be accessed here:

http://www.biomedcentral.com/1755-8794/3/14/prepub

## Supplementary Material

Additional file 1 Clinicopathological features of 24 patients with larynx SCC and of 23 patients with tongue SCC. Click here for file

Additional file 2 Underexpressed proteins in Hep-2 cells and fibroblasts treated with conditioned medium from fibroblasts (FCM) and Hep-2 (HCM), respectively. Click here for file
