# Supplementary material for: Genomics and proteomics approaches to the study of cancer-stroma interactions
Source: BMC Med Genomics. 2010 May 4;3:14. doi: 10.1186/1755-8794-3-14 (PMC2881110; doi:10.1186/1755-8794-3-14)
Supplement: Additional file 1 — Clinicopathological features of 24 patients with larynx SCC and of 23 patients with tongue SCC. [file 1755-8794-3-14-S1.DOC]

**Additional file 1. Clinicopathological features of 24 patients with laryngeal SCC and 23 patients with oral tongue SCC.**

| **Case** | **Site** | **Pathologic stage** | ***ARID4A* expression (Fold change)** | **Histological differentiation** | **Vascular infiltration** | **Lymphatic**  **infiltration** | **Perineural**  **invasion** |
| --- | --- | --- | --- | --- | --- | --- | --- |
| 1 | Tongue | T2N0M0 | -6.94250 | Well | No | No | Yes |
| 2 | Tongue | T2N0M0 | -6.34424 | Well | No |  |  |
| 3 | Larynx | T3N0M0 | -4.83139 | Moderate | No | No | No |
| 4 | Larynx | T4N2BM0 | -4.66025 | Well | No | Yes | No |
| 5 | Larynx | TN1M | -4.27485 | Moderate | No | No | No |
| 6 | Tongue | T1N2BM0 | -4.18699 | Well | No | Yes | No |
| 7 | Tongue | T3N0M0 | -4.07653 | Moderate | No | No | Yes |
| 8 | Larynx | T2N0M | -3.29721 | Moderate | No | No | No |
| 9 | Larynx | T2NM | -3.15294 | Moderate | No | No | Yes |
| 10 | Tongue | T2N2bM0 | -2.75169 | Well | No | Yes | No |
| 11 | Larynx | T4N0M0 | -2.21241 | Moderate | No | No | No |
| 12 | Larynx | T4N2cM0 | -2.20134 | Moderate | No | No | Yes |
| 13 | Tongue | T1N2bM | -2.17014 | Moderate | No | No | No |
| 14 | Larynx | T3N0MX | -2.06172 | Well | No | No | No |
| 15 | Tongue | T3N0M0 | -1.82874 | Moderate | No | Yes | Yes |
| 16 | Larynx | T2N1M0 | -1.74822 | Well | Yes | No | No |
| 17 | Larynx | TN3M | -1.67320 | Poor | Yes | Yes | Yes |
| 18 | Tongue | T2N0M0 | -1.45372 | Moderate | No | No | No |
| 19 | Larynx | T4N0M0 | -1.37537 | Well | No | No | No |
| 20 | Tongue | T2N2bM0 | -1.25641 | Well | No | Yes | Yes |
| 21 | Larynx | T3N3M0 | -1.21541 | Moderate | Yes | Yes | Yes |
| 22 | Tongue | T1N0M0 | -1.21272 | Moderate | No | No | No |
| 23 | Larynx | T4N0M0 | -1.04545 |  | No | No | No |
| 24 | Tongue | T3N0M0 | -0.74004 | Moderate | No | No | Yes |
| 25 | Tongue | T3N0M0 | -0.47105 | Moderate | Yes | Yes | No |
| 26 | Tongue | T4N1M0 | -0.39065 | Well | No | No | No |
| 27 | Larynx | T4N2bM0 | -0.15034 | Moderate | No | No | No |
| 28 | Tongue | T2N0M0 | 0.12950 | Well | No | No | No |
| 29 | Larynx | T3N0M0 | 0.13108 | Moderate | No | No | No |
| 30 | Tongue | T3N0M0 | 0.16402 | Well | No | No | No |
| 31 | Larynx | T2N0M0 | 0.18754 | Well | No | No | No |
| 32 | Tongue | T4N2M0 | 0.19148 | Poor | No | No | No |
| 33 | Larynx | T3N2cMx | 0.19581 | Moderate | No | Yes | No |
| 34 | Tongue | T3N0M0 | 0.20828 | Moderate | No | No | No |
| 35 | Tongue | T2N0M0 | 0.27124 | Moderate | No | No | No |
| 36 | Larynx | T2N0M0 | 0.40117 | Moderate | Yes | No | Yes |
| 37 | Larynx | T3N0M0 | 0.59043 | Moderate | No | No | Yes |
| 38 | Tongue | T4N2M0 | 0.66753 | Poor | No | Yes | Yes |
| 39 | Larynx | Tr4N0M0 | 0.67217 | Moderate |  | Yes | Yes |
| 40 | Larynx | T2N1M0 | 0.84555 | Poor | No | No | No |
| 41 | Tongue | T3N0M0 | 1.51330 | Moderate | No | No | Yes |
| 42 | Larynx | T4N0M0 | 1.71724 | Well | No | No | Yes |
| 43 | Larynx | T3N0M0 | 1.82247 | Well | No | No | No |
| 44 | Tongue | T2N0M0 | 2.13289 | Well | No | No | No |
| 45 | Larynx | T4N2cM0 | 2.20806 | Moderate | No | No | Yes |
| 46 | Tongue | T1N1M0 | 3.30854 | Moderate | No | No | Yes |
| 47 | Tongue | T2N2cM0 | 6.26179 | Moderate | No | No | Yes |
