# Supplementary material for: Genomics and proteomics approaches to the study of cancer-stroma interactions
Source: BMC Med Genomics. 2010 May 4;3:14. doi: 10.1186/1755-8794-3-14 (PMC2881110; doi:10.1186/1755-8794-3-14)
Supplement: Additional file 2 — Underexpressed proteins in Hep-2 cells and fibroblasts treated with conditioned medium from fibroblasts (FCM) and Hep-2 (HCM), respectively. [file 1755-8794-3-14-S2.DOC]

**Additional file 2. Underexpressed proteins in Hep-2 cells and fibroblasts treated with conditioned medium from fibroblasts (FCM) and Hep-2 (HCM), respectively.**

| **Protein** | **SwissProt accession** | **Score*** | **Sequence coverage (%)** | **Biological Process** |
| --- | --- | --- | --- | --- |
| **HEP-2 cells treated with FCM** |  |  |  |  |
| Alpha-enolase | P06733 | 84 | 11 | transcription  growth control  hypoxia tolerance  allergic responses |
| Heterogeneous nuclear ribonucleoproteins C (C1/C2) | P07910 | 85 | 10 | RNA splicing |
| Fructose-bisphosphate aldolase A | P04075 | 143 | 12 | glycolysis |
| Tubulin beta-1 chain | Q9H4B7 | 73 | 9 | cell motion |
| Glyceraldehyde-3-phosphate dehydrogenase | P04406 | 71 | 10 | glycolysis  membrane trafficking |
|  |  |  |  |  |
| **Fibroblasts treated with HCM** |  |  |  |  |
| Vimentin | P08670 | 82 | 5 | cell motion |
| Actin, cytoplasmic 1 | P60709 | 42 | 4 | cell motion |

*Scores greater than 40 were considered significant (p< 0.05).
